# Long-Term Tamoxifen Effects in the Cyclic Interaction of the Endocannabinoid and Endocrine System in the Rat Central Nervous System

**DOI:** 10.3390/biomedicines11030720

**Published:** 2023-02-27

**Authors:** Bruno M. Fonseca, Niloy Bhowmick, Sara Cunha, João Maia, Georgina Correia-da-Silva, Natércia Teixeira, Susana I. Sá

**Affiliations:** 1 UCIBIO—Applied Molecular Biosciences Unit, Departamento de Ciências Biológicas, Laboratório de Bioquímica, Faculdade de Farmácia da Universidade do Porto, 4050-313 Porto, Portugal; 2 Associate Laboratory i4HB—Institute for Health and Bioeconomy Departamento de Ciências Biológicas, Laboratório de Bioquímica, Faculdade de Farmácia da Universidade do Porto, 4050-313 Porto, Portugal; 3 LAQV, REQUIMTE, Departamento de Ciências Químicas, Laboratório de Bromatologia e Hidrologia, Faculdade de Farmácia da Universidade do Porto, 4050-313 Porto, Portugal; 4 Unit of Anatomy, Department of Biomedicine, Faculdade de Medicina da Universidade do Porto, 4200-319 Porto, Portugal; 5 CINTESIS, Center for Health Technology and Services Research, Faculdade de Medicina da Universidade do Porto, 4200-319 Porto, Portugal

**Keywords:** anandamide, estrogen, hypothalamic–pituitary–gonadal axis, cannabinoids, mood, cognition

## Abstract

Steroid hormones can modulate the endocannabinoid system (ECS). Within the female reproductive tract, estrogen increases the expression of the cannabinoid receptors CB1 and CB2, and modifies the levels of anandamide (AEA), the major endocannabinoid, by altering the expression of both AEA synthesis (NAPE-PLD) and catabolic enzymes (FAAH). Here, we addressed the mechanisms involved in ECS fluctuations within the central nervous system and evaluated the effects of tamoxifen (TAM), a selective estrogen receptor modulator, in central AEA regulation. The current results suggest that the hypothalamic and pituitary AEA levels change differently according to the brain area and phase of the estrous cycle. In TAM-treated rats, there is a disruption of the cyclic fluctuation and reduction of the AEA levels in all brain areas. In the pituitary gland, NAPE-PLD expression increases in the metestrus phase, whereas throughout the rat cycle their expression remained constant, even upon TAM treatment. The fluctuations of pituitary AEA levels result from altered FAAH and NAPE-LPD expression. In contrast, no differences in FAAH or NAPE-PLD hypothalamic expression were observed. Overall, this study presents a broad view of the distribution and expression of ECS elements in the central nervous system and a way to suggest possible brain areas involved in the interaction of the endocannabinoid and neuroendocrine systems to induce several behavioral responses.

## 1. Introduction

Cannabinoid receptors together with endocannabinoids and respective metabolic enzymes compose the endocannabinoid system (ECS). The two most well-known endocannabinoids are arachidonic acid derivates—N-arachidonoylethanolamide (anandamide; AEA), and 2-arachidonoylglycerol (2-AG). The ECS plays key modulatory roles in the regulation of a range of physiological processes. In the brain, the ECS is involved in synaptic plasticity and learning [1], motivation and reward [2], emotional and non-emotional memory processes [3], as well as neurogenesis [4]. In turn, dysfunction in endocannabinoid signaling is associated with neurological and mood disorders. Studies of cerebrospinal fluid in acute paranoid-type schizophrenic report higher levels of 2-AG than in healthy controls [5]. Contrary to 2-AG plasmatic concentrations that are lower in unipolar depression, AEA levels may be increased or decreased according to disease stage. In addition, postmortem studies of patients suffering from unipolar depression provide evidence of increased levels of CB1 receptor mRNA along with higher CB1 receptor binding and functionality [6]. In turn, no such differences have been reported in a small sample of bipolar patients [7]. Although the medical use of cannabinoids remains controversial, the approval of cannabinoids for the treatment of spasticity and neuropathic pain in multiple sclerosis, and more recently, the non-psychotropic cannabinoid cannabidiol (CBD) for the treatment of otherwise untreatable severe forms of childhood epilepsy, have brought the therapeutic use of cannabinoids in neurological diseases into the limelight.

Current evidence suggests that the ECS interacts with gonadal hormones. In the female reproductive tissues, estrogen increases the expression of the cannabinoid receptors, CB1 and CB2, and of the AEA metabolic enzymes, the N-acyl phosphatidylethanolamine (NAPE)-specific phospholipase D (NAPE-PLD) [8] involved in the synthesis, and the catabolic enzyme fatty acid amide hydrolase (FAAH) [9], indicating a direct regulatory role in the modulation of ECS [10]. In the female hypothalamus, the AEA levels markedly increase before puberty [11], suggesting a relevant role for AEA in the onset of puberty in rats. By contrast, no consistent differences were observed in AEA levels in the anterior pituitary [11]. In addition, AEA may tonically activate CB1 receptors in the hypothalamus to maintain food intake, whereas food intake inhibitors reduced central AEA levels [12]. Overall, previous studies have demonstrated an interaction between estradiol and AEA in the feedback mechanism for the control of energy intake. Moreover, estrogen modifies AEA levels, affecting its action and modulating ECS components. AEA levels are higher in estrogen-primed ovariectomized (OVX) rats than in OVX and male rats [13]. The interplay between ovarian steroid hormones and endocannabinoids is complex and involves the hypothalamic-pituitary-ovarian axis. The plasma AEA levels correlates with luteinizing hormone, follicle-stimulating hormone and estradiol plasma levels [14]. In addition, in OVX rats, the administration of AEA increased plasma prolactin concentrations [15] and decreased luteinizing hormone-releasing hormone (LHRH) followed by decreased luteinizing hormone release [13], a clear indication of the combined action of the endocrine and ECS.

Since the modulation of the enzymes responsible for AEA metabolism and, consequently, of AEA levels in female rats are modulated by estradiol fluctuation, it may highlight the normal fluctuations of AEA in the hypothalamus and pituitary throughout the reproductive cycle. A better characterization of the mechanisms involved in these fluctuations in the neuroendocrine areas, namely the pituitary and the preoptic and hypothalamic areas (POA-Hyp area) could contribute to the understanding of central AEA regulation of mood and emotion, which could contribute to the development of new therapeutic alternatives to mood disorders. Still, the widespread expression of the ECS components in the areas that modulate the neuroendocrine system and the possible endocannabinoid regulation of such system requires further elucidation.

Tamoxifen (TAM) is a selective estrogen receptor (ER) modulator used as an endocrine therapy for pre- and postmenopausal women with ER-positive breast cancer [16,17,18]. As it substantially improves disease-free survival, the national and international guidelines establish TAM therapy for 5-10 years for premenopausal women with estrogen receptor (ER)-positive breast cancer [16,17,18]. Despite the benefits to the quality of life and the economic burden of cancer, clinical trials indicate that TAM action through ER activation induces mood disorders, such as depression, anxiety and irritability, and impair cognitive performance [19,20,21]. The POA-Hyp area, namely the preoptic area (POA), the lateral hypothalamic area (LH), the hypothalamic ventromedial (VMN) and dorsomedial nuclei (DMN), is crucial in the regulation of motivated behaviors, such as food consumption, parental care, and of emotional responses, such as anxiety and depression (mood disorders) via the hypothalamic-pituitary-gonad or hypothalamic-pituitary-adrenal axis [22,23,24]. These brain areas express abundantly ERs and it is known that the action of estradiol through ERs in these areas affect the connectivity pathways involved in depression and anxiety, which is the main reason for the lack of protection upon menopause. We have previously observed that TAM binding to ERs in the POA-Hyp area, mimics the menopausal effects, being able to disturb female endocrine response, affecting peripheral organs and VMN neuronal physiology [25]. By studying the effects of TAM in the modulation of the ECS components in the preoptic area and hypothalamus, brain areas known for their vulnerability to mood disorders, we aim to assess the effects of ER activation in the ECS components expression and to correlate with possible role of this system activation in the protection of this brain circuitry.

With the present study, we aim to determine the effects of long-term TAM therapy in the ER-dependent expression of ECS components. By determining the fluctuation levels of ECS components along the fluctuations of estradiol levels in the preoptic area and hypothalamus, and by assessing the effects of TAM modulation of ERs in this mechanism, we aim to devise a possible venue of action of TAM/ECS modulation of the pathways involved in mood disorders and cognition. For this, an animal model of long-term TAM therapy was used [26].

## 2. Materials and Methods

### 2.1. Animal Care and Use

Three-month-old, young cycling Wistar female rats (I3S, Porto, Portugal), maintained under standard laboratory conditions (12 h dark/light cycle, lights on at 7:00 am, and room temperature of 23 °C), with free access to standard solid diet and tap water, were randomly assigned to the treatment and control groups (final n  =  10 per group). The treatment group received 50 µL of a solution containing TAM (5 mg/kg/day) in 0.5% hydroxypropylmethyl cellulose (HMC) mixed in hazelnut chocolate (150 mg), p.o., daily, for 3 months (TAM-group), at 2:00 pm. Age- and body weight-match control rats received the same amount of HMC solution in the same amount of hazelnut chocolate. TAM dose and schedule of administration was determined in a pilot study and then applied in a previous study from our lab [26,27]. The dose was chosen based on the allometric scaling of the human/rat equivalent dose and by comparing physiological and metabolic responses in rat and humans taking TAM [26,27]. Estrous cycle was monitored daily by vaginal smear cytology, performed at 2:00 pm. Food and water intake and body weight were regularly recorded. Both TAM and the vehicle were purchased from Sigma-Aldrich Company Ltd. (Madrid, Spain).

### 2.2. Tissue Processing

At the end of the experimental period, half of the rats were assigned for morphological studies (n = 5 per group) and the other half for biochemical determinations (n = 5 per group). Animal number per group was determined according to previously performed power analysis (n = 5 > power sample of 0.8 and type I error rate of 0.05).

**Figure 1:**
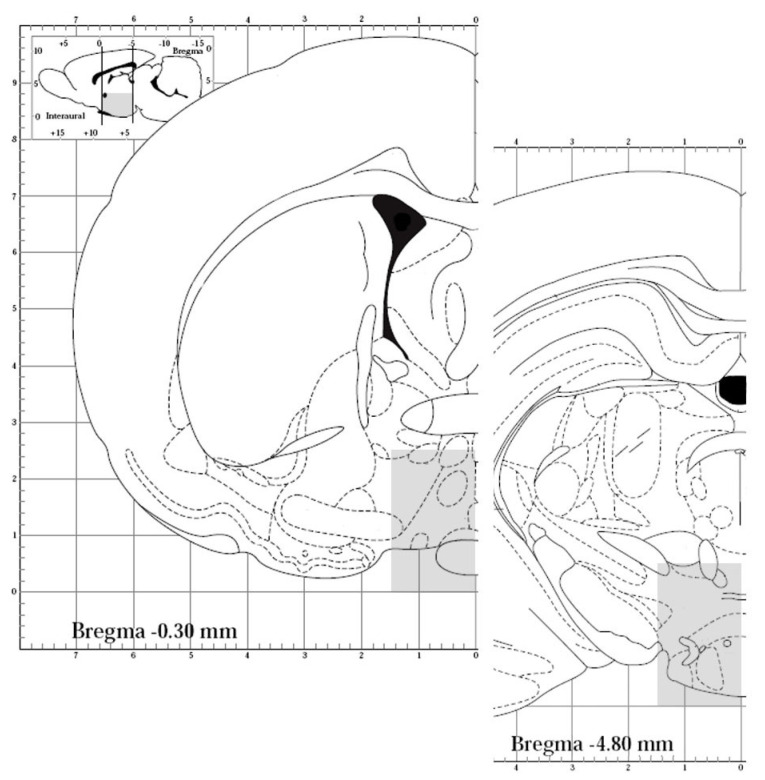
Representative drawing of the POA-Hyp (preoptic-hypothalamic) area dissected for biochemical studies. The dissecting planes were made between the anterior border of the optic chiasm and the posterior border of the mammillary bodies (aprox. Bregma −0.50 to −5.00); 1.5 mm lateral to the midline and 3.5 mm dorsal to the ventral base. The area is represented by the gray shaded areas in a representative section of the rostral and of the caudal levels (adopted from http://labs.gaidi.ca/rat-brain-atlas, accessed on 1 September 2022).

Rats used for immunohistochemical studies were anesthetized with sevoflurane and sacrificed by intracardiac perfusion with 4% paraformaldehyde in 0.1 M phosphate buffer, pH 7.6, between 2:00 and 3:00 pm of the day of the required estrous phase, in order to match smear cytology. Since TAM was shown to abolish ovarian hormone fluctuations [28], TAM-treated rats were sacrificed at the same time point and with the normally cycling animals. The pituitary and brain of all animals were removed from the skulls, weighed, and immersed in the same fixative solution for 1 h, before processing. Animals used for biochemical studies were sacrificed by decapitation, at the same time pint, upon sevoflurane anesthesia; the pituitaries and brains were then dissected and the right and left POA-Hyp area were isolated and processed together in all biochemical analyses. The POA-Hyp area includes the preoptic area and hypothalamus. The dissecting planes were made between the anterior border of the optic chiasm and the posterior border of the mammillary bodies (aprox. Bregma −0.50 to −5.00); 1.5 mm lateral to the midline and 3.5 mm dorsal to the ventral base (Figure 1). In all sacrifices, blood was collected from the heart, centrifuged (3000× *g*; 4 °C; 10 min), and plasma was separated and stored at−80 °C before analysis.

### 2.3. Immunohistochemistry Processing and Qualitative Analysis of Pituitary

**Figure 2:**
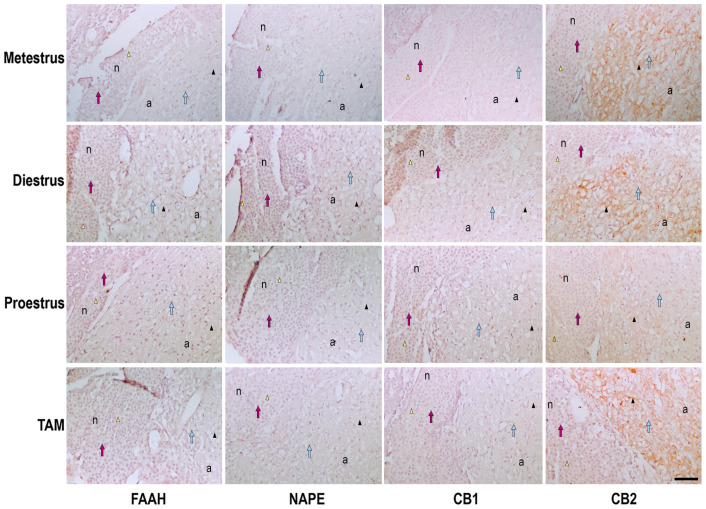
Photomicrograph representation of the immunohistochemical detection and cellular distribution of FAAH, NAPE, CB1 and CB2 in the pituitary of metestrus, diestrus and proestrus rats and cycling female rats treated with TAM. n, neurohypophysis; a, adenohypophysis; arrow heads, positive immunostaining in neurohypophysis (yellow) or adenohypophysis (black); arrows, cells in neurohypophysis (pink) or adenohypophysis (blue). Scale bar = 100 μm.

The pituitary was embedded in paraffin and sectioned. The 5 µm-thick sections were mounted on poly-L-lysine-coated slides. From each rat, one set of slides was stained with hematoxylin & eosin, and another was processed for immunohistochemistry, as previously described [10]. Briefly, after dewaxing and rehydration, antigen recovery was performed with citrate buffer. Then, sections were incubated with sodium borohydride for 30 min, followed by hydrogen peroxide for 10 min. Subsequently, slides were incubated overnight at 4 °C, with the primary antibodies against CB1, CB2, NAPE-PLD and FAAH (Table 1), all from St. Cruz Biotechnology (Santa Cruz, CA, USA). After washing, they were incubated with diluted biotinylated secondary antibodies (Table 1) for 1 h, followed by incubation with avidin-biotin peroxidase complex (Vectastain Elite ABC Kit) from Vector Laboratories (Burlingame, CA, USA). The reaction was developed by incubation with 3,3′-diaminobenzidin (DAB). Negative controls were performed with the inclusion of blocking serum instead of primary antibodies or by using the corresponding blocking peptide. The slides were counterstained with Mayer’s hematoxylin solution (Sigma-Aldrich company Ltd., Madrid, Spain), mounted in Entelan mounting medium (Merck, Darmstadt, Germany) and then used for the qualitative analyses of immunohistochemistry and cellular distribution of each enzyme and receptor (Figure 2). The semi-quantitative analyses were carried out by two different observers blinded to group assignment, using a 10× objective of a Nikon microscope (Nikon Eclipse Ci-L) in a set of 4 sections per rat, making a total of 20 section per group. The density of expression of ECS enzymes and receptors (Table 2) was qualitatively classified as rare (+/−, low (+), moderate (++) or high (+++), according to the density of labelling.

### 2.4. Immunohistochemistry Processing and Qualitative Analysis of Hypothalamus

Brains were transected in the coronal plane through the anterior border of the optic chiasm, rostrally, and the posterior limit of the mammillary bodies, caudally. The blocks were mounted on a Vibratome, sectioned at 40 μm, and sampled at regular intervals of 120 μm (one out of three sections), throughout the rostro-caudal extent pre-optic-hypothalamus area. Immunostaining was carried out as previously described in detail [10,29,30] using the primary antibodies against CB1, CB2, NAPE-PLD and FAAH (Table 1), all from St. Cruz Biotechnology (CA, USA) and incubated for 72 h, at 4 °C. After washing, the sections were incubated with the respective biotinylated secondary antibody (Table 1) followed by the ABC Kit. The staining was detected with DAB. After washing, stained sections were mounted on gelatin-coated slides, air-dried, dehydrated and cover-slipped using Histomount (National Diagnostics, Atlanta, GA, USA).

**Figure 3:**
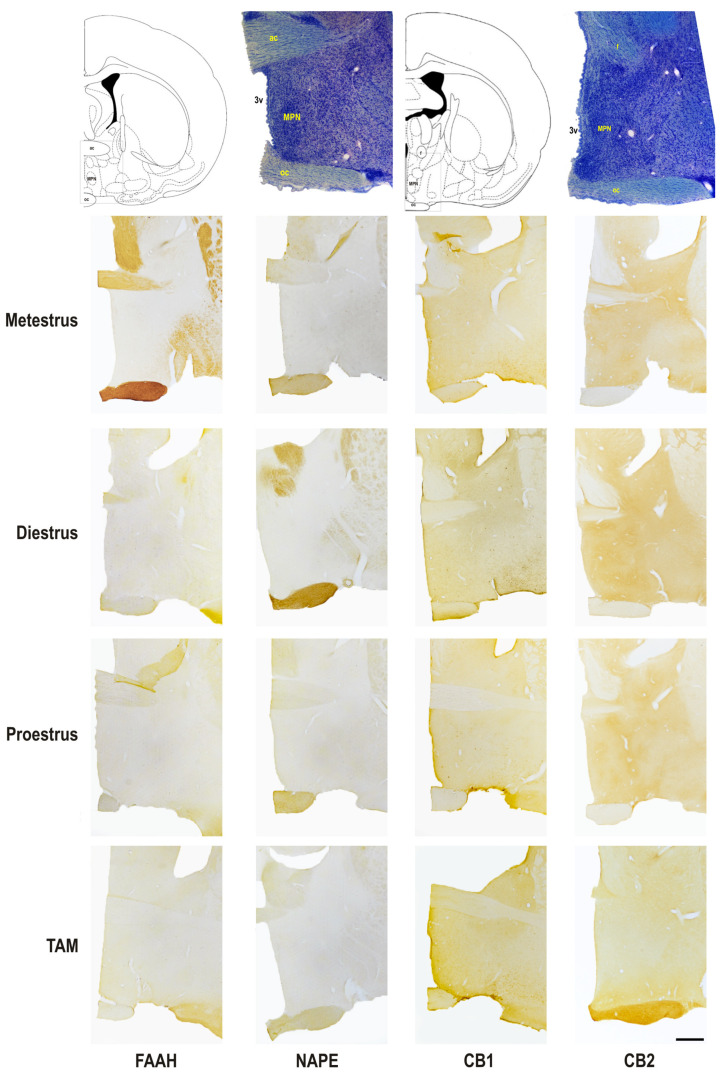
Photomicrograph representation of the immunohistochemical detection and cellular distribution of FAAH, NAPE, CB1, and CB2 in the medial preoptic nucleus of metestrus, diestrus and proestrus rats and cycling female rats treated with TAM. On top: line drawing and giemsa staining sections identifying two rostro-caudal levels of the area, at Bregma level −0.40 and −0.92. 3v, third ventricle; ac, anterior commissure; f, fornix; MPN, medial preoptic nucleus; oc, optic chiasma. Scale bar = 500 μm.

**Figure 4:**
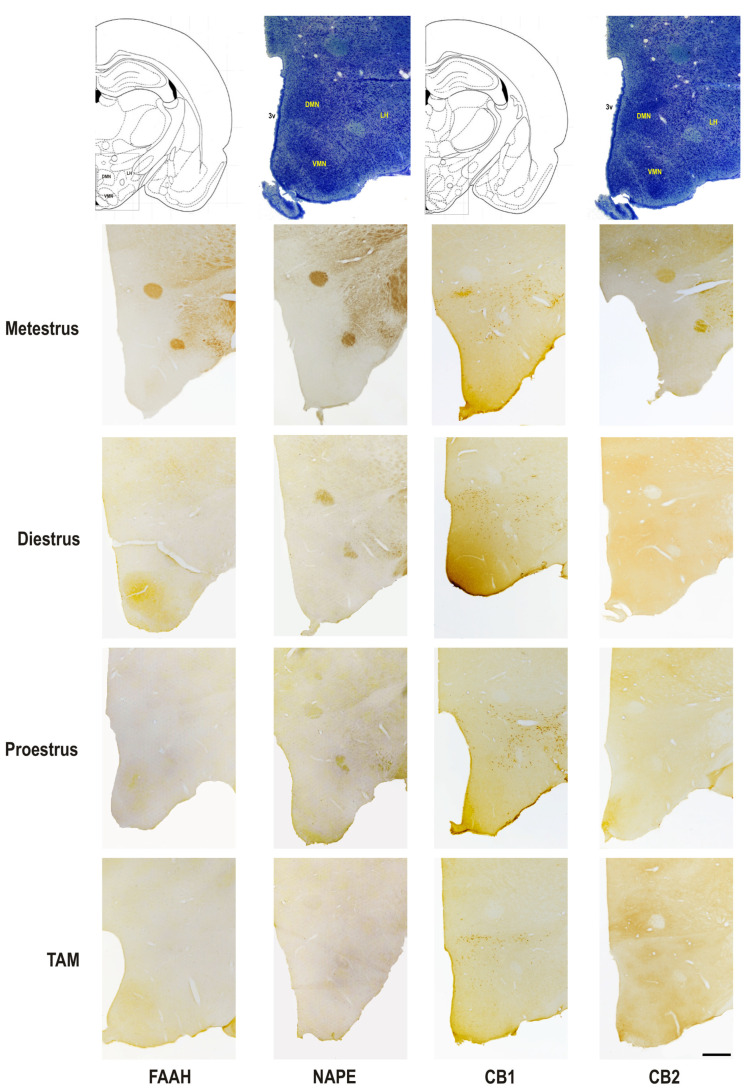
Photomicrograph representation of the immunohistochemical detection and cellular distribution of FAAH, NAPE, CB1, and CB2 in the lateral hypothalamic area, the hypothalamic ventromedial and dorsomedial nuclei of metestrus, diestrus and proestrus rats and cycling female rats treated with TAM. On top: line drawing and giemsa staining sections identifying two rostro-caudal levels of the areas, at Bregma level −3.14 and −3.30. 3v, third ventricle; DMN, dorsomedial nucleus of the hypothalamus; LH, lateral hypothalamic area; VMN, ventromedial nucleus of the hypothalamus. Scale bar = 500 μm.

For the semi-qualitative analyses of immunohistochemistry and cellular distribution of each enzyme and receptor evaluated in the medial preoptic (MPN), lateral hypothalamic area (LH), the hypothalamic ventromedial (VMN) and dorsomedial nuclei (DMN), rostro-caudal extension was examined by two different observers blinded to group assignment, using a 10× objective of a Nikon microscope (Nikon Eclipse Ci-L). The analysis was carried out in about 2-3 sections through the preoptic area (MPN, Figure 3) and 3-4 sections through the hypothalamus (LH, VMN and DMN, Figure 4), per rat. The fiber and neuron density (Table 3) was qualitatively classified as rare (+/−), low (+), moderate (++) or high (+++) according to the density of labelling.

### 2.5. Western Blotting

Hypothalamus or pituitary tissue was homogenized using pellet pestle in lysis buffer (20 mM HEPES, 2 mM EDTA, 10 mM KCl, 1.5 mM MgCl_2_) supplemented with protease inhibitor. After centrifugation of the protein extracts at 700 *g* for 10 min, the supernatant was collected and the protein concentration was determined by the Bradford method. Protein extracts (25 μg) were separated by 10% SDS–PAGE and transferred to nitrocellulose membrane in a semi dry transfer system (Trans-Blot SD semi dry transfer cell, Bio-Rad). Primary antibodies (NAPE-PLD and FAAH; Table 1) were incubated overnight at 4 °C in blocking solution (5% of dry milk in phosphate buffer (PBS) with Triton X-100, 0.1%). Secondary antibody was horseradish peroxidase-linked and the membranes were exposed to chemiluminescent substrate Super Signal West Pico and immunoreactive bands were visualized by ChemiDocTM Touch Imaging System (BioRad, Laboratories Melville, NY, USA). The membranes were then stripped and incubated with anti-β-actin (Table 1, Santa Cruz Biotechnology, CA, USA) to control loading variation.

### 2.6. UPLC-MS/MS Method for AEA Quantification

The POA region of hypothalamus and pituitary gland were used for analysis of AEA concentrations by using liquid chromatography coupled to tandem mass spectrometry (UPLC-MS/MS). Briefly, tissues were weighed and spiked with 2 μL of the internal standard AEA-d4 (2.8 μM; Cayman Chemicals, Ann Arbor, MI, USA) and submitted to a two cycle of 20 s with 5 s in-between homogenization with 1 g of zirconium beads and 500 μL of toluene performed in a Precellys 24 homogenizer (Bertin Technologies, France). After centrifugation at 5000× *g* for 5 min at 4 °C, the upper phase was collected, dried at constant nitrogen stream, and then reconstituted in 100 μL of phase B (96% methanol (MeOH) and 4% ammonium acetate (AA) 2 mM) and transferred to an HPLC vial ready for UPLC-MS/MS analysis.

Plasma (500 μL) was also spiked with deuterated internal standard solution and 0.4 g of MgSO_4_, 0.1 g NaCl and 500 μL of ethyl acetate were added to each sample followed by a vortex homogenization and centrifugation at 3500× *g* for 10 min at room temperature. The upper phase was collected, dried at constant nitrogen stream, reconstituted in phase B following UPLC–MS/MS analysis. Chromatographic and detection details were reported in a previous work [10].

### 2.7. Statistical Analysis

All numerical data are expressed as mean ± SEM. Statistical analysis was performed to the biochemical data using one-way ANOVA, followed by Bonferroni *ad hoc* post-test to make pairwise comparisons of individual means (GraphPad PRISM version 6.0; GraphPad Software, Inc., San Diego, CA, USA) when significance was indicated. Correlation between the plasmatic hormone levels and tissue AEA levels were calculated using Spearman rank-order correlation. Differences were considered to be statistically significant when *p* < 0.05. All of the assays were performed in triplicate in, at least, three independent experiments.

## 3. Results

### 3.1. Fluctuations of AEA Levels and AEA-Metabolic Enzymes on Pituitary and POA-Hyp

**Figure 5:**
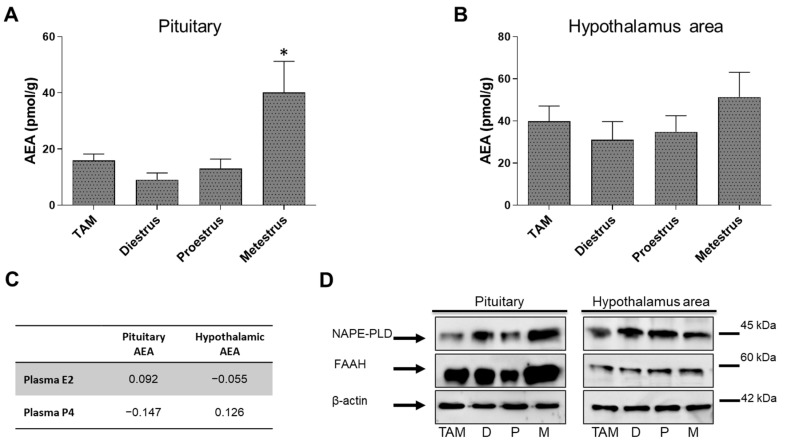
Graphical representation of the AEA levels measured by HPLC in the whole pituitary (**A**) and in the POA-Hyp (preoptic-hypothalamic) area (**B**) of metestrus, diestrus and proestrus rats and cycling female rats treated with TAM. (**C**) Spearman’s rank correlations coefficient between plasmatic hormone and AEA levels. (**D**) NAPE-PLD and FAAH levels measured by Western blot in the whole pituitary and in the POA-Hyp area of metestrus, diestrus and proestrus rats and cycling female rats treated with TAM. Bonferroni ad hoc post-test: * *p* < 0.05, compared with all other groups.

The AEA levels were measured in pituitary and POA-Hyp areas of cycling female rats, as well as in TAM treated animals by UPLC-MS/MS (Figure 5). Results show differences in AEA levels only in the pituitary, with metestrus rats presenting the highest levels (Figure 5A). We have previously measured estradiol (E2 and progesterone levels (P4) [28]. When correlated, no significant relationship was observed between plasma hormone and AEA tissue levels (Figure 5C). In pituitary, NAPE-PLD expression increased in metestrus rats, while FAAH expression decrease (Figure 5B,D). Regarding the POA-Hyp, the results show a constant FAAH and NAPE-PLD expression throughout the cycle. TAM treatment did not induce any alterations in both enzyme expression (Figure 5D).

### 3.2. Semi-Quantitative Analysis of Protein Expression in the Pituitary

At diestrus, the expression of the enzymes FAAH and NAPE-PLD increase while CB1 expression decreases. However, an increase in CB1 expression is observed at proestrus and TAM decreased its expression when compared to the latter phase. In addition, TAM administration reduces the expression of FAAH and NAPE-PLD, when compared with diestrus rats, but increases the expression of CB2 to values such as diestrus in adenohypophysis (Table 2). Besides CB2, which was expressed in higher levels in adenohypophysis, the expression of the other ECS elements was similar between neurohypophysis and adenohypophysis.

### 3.3. Semi-Quantitative Analysis of Protein Expression in the Pre-Optic Area and Hypothalamus

Results show that in the analyzed brain areas, CB receptors are more expressed than the metabolic enzymes of the ECS, with NAPE-PLD as the least expressed component. In addition, in the VMN, animals at proestrus presented the lower expression levels of FAAH enzyme.

In the VMN, the expression of FAAH is lower at proestrus, but is higher than in the other areas throughout the cycle. NAPE-PLD expression is similar throughout the cycle in all areas, though higher levels were detected at metestrus. The expression of CB1 is highest in the MPN and LH and increases to identical levels in VMN in diestrus cycle (Table 3). The expression of CB2 increases at diestrus in the DMN and VMN and decreases at diestrus in the LH (Table 3). TAM administration increases CB1 and reduce the expression of CB2, being lower in DMN, VMN, and LH. TAM administration also increases the expression of FAAH in the MPN, while NAPE-PLD expression is similar to the one observed throughout rat cycle in all analyzed brain areas (Table 3).

## 4. Discussion

Endocannabinoids modulate neurotransmitter release at both excitatory and inhibitory synapses through a retrograde signaling mechanism that involves activation of cannabinoid type 1 (CB1) receptor. While CB1 is mainly expressed in the central nervous system [30], CB2 receptor is mostly found in the periphery, particularly at immune cells [31]. The AEA levels in the brain appear to be tightly controlled as the selective inhibition of FAAH produces dramatic increases in AEA levels in brain tissue, resulting in hypoalgesia [32] and altered behavioral phenotypes [33]. It points to FAAH inhibition as a viable pharmacological approach to treat numerous central nervous system disorders including neurodegenerative diseases, mood disorders, and cognitive deficits, among others. Another line of evidence points to AEA interaction with steroid hormones. The ECS is modulated by estradiol. FAAH presents an estrogen response element that, when activated by ERs, inhibits its transcription and, thus, increases AEA levels [34].

Current data suggest that ECS levels change differently according to brain area and phase of the estrous cycle, which matches the known effect of steroid hormones in the modulation of the ECS [14,35]. In the pituitary, metestrus rats present higher levels of AEA, suggesting, in accordance with previous studies, that progesterone plays a key role in the modulation of the ECS. In fact, metestrus is the phase of the estrous cycle characterized by increased progesterone levels from the corpus luteum, without concomitant increase in estradiol [36], and although not in a significant way, also shows the impact of progesterone levels in the studied brain areas, as already suggested [37,38,39]. The frontal lobe bears the Accumbens nucleus and the brain area responsible for the rewording feeling and addiction [40,41]. AEA exerts an overall modulatory effect on the reward circuitry [42]. The major change in TAM-treated rats is the disruption of the cyclic fluctuation, has we have previously shown [10,27]. The disruption of cyclic fluctuations induced by changes in sex steroid levels are more relevant for the female brain than the continuous high or low hormone levels [43,44]. Both estrogen and progesterone levels fluctuate along the estrous cycle, and this will induce changes in the functioning of the CNS. Thus, the ECS can fluctuates with and due to estrogen and progesterone levels, inducing its effects in the central and peripheral physiology. In a previous study of our group [28], we have found that TAM, in the present dose and schedule of administration, disrupts estradiol and progesterone cyclicity. These past results combined with present ones lead to the suggestion that, by affecting the cyclic fluctuation of ovarian hormones, TAM is able to affect endocannabinoids action, triggering a possible disruption mechanism of behavioral responses.

In addition, the present results suggest that the fluctuations of pituitary AEA levels are due to changes in the levels of FAAH and NAPE-PLD. The expression of both enzymes is similar between the endocrine (adenohypophysis) and neuronal (neurohypophysis) parts of the gland, although its expression is scattered along the entire anterior pituitary. The fluctuation in FAAH expression in the hypothalamus is similar throughout the cycle in the four areas analyzed by semi-quantitative analysis. The enzyme is most expressed in the VMN where it is mostly present in fibers. The increased expression of FAAH at metestrus reflects changes in the VMN, being indicative of the increased levels of AEA, due to the degradation action of the enzyme. Although the Western blot shows NAPE expression in the hypothalamus area, its expression is rare and difficult to identify by immunohistochemical detection. The fluctuations seem to account for the fluctuations in the LH, the area that most expresses the enzyme. The results also show that CB1 is mostly expressed in the MPN and LH, areas related with regulation of anxiety and locomotor activity [45,46,47,48]. The VMN is known to be the place for modulation and triggering of the receptive component of the female sexual behavior [45,47], and the area where there is regulation of ECS activity by estradiol through modulation of CB receptors. Interestingly, present results indicate that CB1 is expressed mostly in VMN fibers and not so much in its neurons. Since the MPN and VMN have been shown to act sequentially in the modulation of female sexual behavior, the presence of CB receptors in VMN fibers and its known regulation by estradiol may be a mechanism for AEA action in the modulation of the behavior.

Overall, the present study agrees with previous ones, being a largely view of the distribution and expression of endocannabinoid system elements in the CNS. The seen neuronal distribution suggests a possible involvement of preoptic and hypothalamic areas in the interaction of the endocannabinoids and neuroendocrine systems that could induce several behavioral responses. The study limitation was the lower number of animals and the large amount of brain tissue used in the biochemical studies, involving dissimilar neuronal areas, which have hampered a powerful statistical significance.

## 5. Conclusions

The present study agrees with previous ones, being a largely view of the distribution and expression of endocannabinoid system elements in the CNS. The seen neuronal distribution suggests a possible involvement of preoptic and hypothalamic areas in the interaction of the endocannabinoids and neuroendocrine systems that could induce several behavioral responses. The study limitation was the lower number of animals and the large amount of brain tissue used in the biochemical studies, involving dissimilar neuronal areas which have hampered a powerful statistical significance.

## Figures and Tables

**Table 1:** Primary and secondary antibodies used.

Antibody Name	Antibody ID (RRID)	Host	Catalog Number	Used Dilution (Brain)	Used Dilution (Pituitary)	Used Dilution (WB)
CB1 (N-15)	AB_637711	Goat	sc-10066	1:1500	1:200	1:200
CB2 (H-60)	AB_2082784	Rabbit	sc-25494	1:1000	1:200	1:200
FAAH (V-17)	AB_2231531	Goat	sc-26427	1:1000	1:100	1:200
NAPE-PLD (G-13)	AB_10841204	Goat	sc-163117	1:1000	1:100	1:200
β-actin (C4)	AB_2714189	Mouse	sc-47778	--	--	1:500
Anti-Goat IgG (H+L)	AB_2336126	Rabbit	BA-5000	1:400	1:150	1:2000
Anti-Rabbit IgG	AB_2313606	Goat	BA-1000	1:400	1:150	1:2000

**Table 2:** Semi-quantitative analysis of staining and distribution of each enzyme and receptor in the pituitary of cycling rats at proestrus and diestrus and of TAM-treated rats.

		Neurohypophysis	Adenohypophysis
FAAH	Metestrus	++	++
	Diestrus	+++	+++
	Proestrus	++	++
	TAM	++	++
NAPE	Metestrus	+	+
	Diestrus	++	++
	Proestrus	+	+/−
	TAM	+/−	+
CB1	Metestrus	+	+
	Diestrus	+/−	+/−
	Proestrus	++	++
	TAM	+	+
CB2	Metestrus	+	+++
	Diestrus	+/−	+++
	Proestrus	+	++
	TAM	+	+++

Density of expression classified as rare (+/−), low (+), moderate (++) or high (+++).

**Table 3:** Semi-quantitative analysis of staining and distribution of neurons and fibers of each enzyme and receptor in the MPN, DMN, VMN and LH of cycling rats at proestrus and diestrus and of TAM-treated rats.

		MPN	DMN	VMN	LH
FAAH	Metestrus	+/−	+/−	++	+
	Diestrus	+	+/−	++	+/−
	Proestrus	+	+/−	+	+/−
	TAM	++	+/−	++	+/−
NAPE	Metestrus	+	+	+	+
	Diestrus	+/−	+/−	+/−	+/−
	Proestrus	+/−	+/−	+	+
	TAM	+/−	+	+/−	+/−
CB1	Metestrus	++	+	+	++
	Diestrus	+	+	++	++
	Proestrus	++	+	+	+
	TAM	++	++	+	+++
CB2	Metestrus	++	+	+	++
	Diestrus	++	++	++	+
	Proestrus	+	+	++	++
	TAM	++	+/−	+/−	+/−

Density of expression classified as rare (+/−), low (+), moderate (++) or high (+++). MPN, medial preoptic nucleus; DMN, dorsomedial nucleus of the hypothalamus; VMN, ventromedial nucleus of the hypothalamus; LH, lateral hypothalamus.

## Data Availability

The data that support the findings of this study are available from the corresponding author upon reasonable request.
